# P-1840. Implementing Universal Viral Hepatitis C Screening within an Emergency Department: Trends from a South Texas Large Urban County Hospital

**DOI:** 10.1093/ofid/ofaf695.2009

**Published:** 2026-01-11

**Authors:** Racquel Owino, Delana Gonzales, Serena Gaultier -Soliz, Dianita Gamino, Makayla Tey, Tatiana Emanuel, Ralph Riviello, Anna Taranova

**Affiliations:** University Health, San Antonio, TX; University Health, San Antonio, TX; University Health, San Antonio, TX; University Health, San Antonio, TX; University Health, San Antonio, TX; University Health Science Center San Antonio, San Antonio, Texas; University Health Science Center San Antonio, San Antonio, Texas; University Health, San Antonio, TX

## Abstract

**Background:**

University Health, is South Texas’ only safety net hospital system serving indigent population within a 28 county region. The Emergency Department (ED) supports infectious disease screening as the last resort for healthcare in the region. Hepatitis C is the most common blood-borne infection, with the strongest risk factor being intravenous drug use. Early detection decreases transmission risk through percutaneous blood exposure. University Health provides universal viral hepatitis C testing within the Emergency Department.

**Methods:**

University Health implemented HCV screening for adults 18 years and over as per Centers for Disease Control and Prevention screening guidelines. A Best Practice Advisory in electronic Medical Record (EMR) system prompts providers to order reflex tests for confirmatory hepatitis C for all adults 18+ that visit the ED. Patients are screened for the virus unless they opt out of testing. Upon delivery of results by a provider, PNs facilitate linkage to care by educating patients and scheduling a follow-up appointment with a primary care physician or specialist.

**Results:**

Between March 2024 to March 2025, 20,713 patients were screened. Of these, 7 %( n = 1,366) had a positive antibody result, of these 89% (n=1,211) received RNA testing. Of all screened, 4% (n= 774) were confirmed positive with 42% (n=326) having a history of HCV, 58% (n=448) are newly diagnosed or status unknown. Of confirmed positive, 17% (n=132 were linked to care). Of the positive, 40% (n=294) had a history of drug usage.

**Conclusion:**

Implementation of universal HCV screening, integrated within the EMR, is crucial to timely identification of infections within the ED. Hepatitis C rates in the region is high, indicating need for opt out ED screening to reach population at highest risk for infectious diseases such as HCV, as a best practice model in identifying gaps in health care provision within a large urban county hospital.

**Disclosures:**

All Authors: No reported disclosures

